# Normal Variations of Sphenoid Sinus and the Adjacent Structures Detected in Cone Beam Computed Tomography

**Published:** 2016-03

**Authors:** Azadeh Rahmati, Roshanak Ghafari, Maryam AnjomShoa

**Affiliations:** 1 Dept. of Oral Radiology, School of Dentistry, International Branch University, Guilan, Iran.; 2 Dept. of Anatomical Science, School of Dentistry, Isfahan (Khorasgan) Branch, Islamic Azad University, Isfahan, Iran.

**Keywords:** Sphenoid Sinus, Internal Carotid Artery, Optic Nerve, Pneumatization, Cone Beam Computed Tomography

## Abstract

**Statement of the Problem:**

The sphenoid sinus is a common target of paranasal surgery. Functional endoscopic sinus surgery is likely to endanger the anatomic variations of vital structures adjacent to the sphenoid sinus.

**Purpose:**

The aim of this study was to determine the variations of sphenoid sinus and the related structures by using cone-beam computed tomography (CBCT).

**Materials and Method:**

In this descriptive-analytic study, CBCT images of 103 patients aged above 20-years were selected (206 sides). Degree of pneumatization of sphenoid sinus, pneumatization of the anterior clinoid process, pterygoid process, protrusion of optic canal, vidian canal, and foramen rotundum, as well as prevalence of sinus septa were recorded. Examinations were performed using On-Demand software (Version 1); data were analyzed by using chi-square test.

**Results:**

There was a statistically significant correlation between the pterygoid pneumatization and vidian canal protrusion (*p*< 0.001), and foramen rotundum protrusion (*p*< 0.001). The optic canal protrusion was found to be significantly associated with the anterior clinoid pneumatization and pterygoid process (*p*< 0.001). Statistically significant relationship was also observed between the carotid canal protrusion and pterygoid process pneumatization (*p*< 0.001).

**Conclusion:**

The anatomical variations of the sphenoid sinus tend to give rise to a complexity of symptoms and potentially serious complications. This variability necessitates a comprehensive understanding of the regional sphenoid sinus anatomy by a detailed CBCT sinus examination.

## Introduction

Being deeply located within the skull, the sphenoid sinus is the most inaccessible paranasal sinus.[1-4] It is surrounded by vital structures, such as the internal carotid artery (ICA), optic nerve (ON), and vidian canals (VC).

Functional endoscopic sinus surgery (FESS), like the conventional sinus surgery, is associated with serious risks. Knowing more about the variable regional anatomy of the sphenoid sinus definitely decreases the surgical complications that follow trans-sphenoidal and functional endoscopic sinus surgery.[1-5] Sphenoid sinus is known as being the most variable cavity of the human body which makes it difficult to approach.[5]

Its pneumatization ranges from absent to extensive,[6] which subsequently makes the bone that covers the carotid arteries, the optic nerves, and the vidian nerves to be thin or even missing. Therefore, the mentioned structures become vulnerable to iatrogenic injury.[7] Accordingly, safe access to the sella is notably influenced by the pattern of pneumatization in the sphenoid sinus.[8] Moreover, anatomic variations are likely to predispose the sphenoid sinus to recurrent or chronic sinusitis.[9]

Hewaidi and Omami[10] reported a statistically significant association between the anterior clinoid process (ACP) pneumatization and ICA protrusion, ACP pneumatization and ON protrusion, pterygoid process (PP) pneumatization and vidian nerve (VN) protrusion. In another study, Kazkayasi *et al.*[11] concluded that VC protrusion was commonly concurrent with pneumatization of the PP. They found a statistically significant correlation between the PP and VC protrusion, and foramen rotundum. The optic canal (OC) protrusion was found to be significantly associated with the ACP; however, no statistically significant correlation existed between CC protrusion and ACP. Sirikci *et al.*[12] noted a significant relationship between the ACP pneumatization and protrusion of the ON into the sphenoid sinus.

Currently, the increasing use of cone-beam computed tomography (CBCT) in placement of dental implants, treatment of sinuses and many other similar instances has helped determining all the relevant anatomic structures and answering the questions of different anatomical variants. In spite of the complex anatomy and vital surgical relationships of the sphenoid sinus, the number of relevant studies using CBCT is very limited. So, the current study was designed to assess variations of sphenoid sinus and the related structures by using CBCT. 

## Materials and Method

This descriptive-analytical study was performed on CBCT of 103 patients that referred for sinus imaging examination, at one of the oral and maxillofacial radiology centers in Isfahan, Iran, from January to August 2014. The participants included 53 males with the mean age of 49 years and 50 females with the mean age of 37.9. Those who had trauma, prior surgery of the sphenoid sinuses, sinonasal tumors, and nasal polyposis were excluded from the study. Patients younger than 20 years of age were also excluded because according to Gray, although the extension of nasal cavity into the body of sphenoid bone -that results in sphenoid sinus- exists before birth, it is likely to reach its full extension only after adolescence.[13]

**Figure 1:**
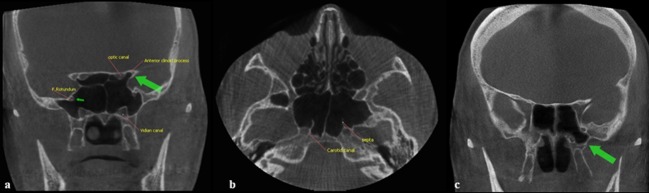
a: Coronal CBCT image: pneumatization of the bilateral anterior clinoid processes and protrusion of optic canal (note that the protrusion of vidian canal and foramen rotundum is bilateral), b: Axial CBCT image: pneumatization of the carotid canal and the septa inserting into its bony covering,  c: Coronal CBCT scan showing the pneumatization of the left pterygoid process (arrow) of the sphenoid sinus

A detailed analysis of CBCT scans of the paranasal sinuses was reviewed by two oral and maxillofacial radiologists with consensus. Bony anatomic variations such as pneumatization of the PP and ACP, protrusions of each of the CC, optic canal (OC), VC or foramen rotundum (FR) into the sinus were recorded (Figures 1). 

The prevalence of these variations was also compared between males and females. If the pneumatization extended below a plane between VC and FR, it was regarded as PP pneumatization. With respect to the degree of pneumatization, the sphenoid sinuses were categorized into four types, depending on the position of the sinus in relation to the sella turcica as Type I: Conchal (completely missing or minimal sphenoid sinus), Type II: Presellar (posterior wall of sphenoid sinus is in front of the anterior wall of the sella), Type III: Sellar (posterior wall is between the anterior and posterior wall of the sella), Type IV: Postsellar (posterior wall of sphenoid sinus is behind the posterior wall of the sella).

**Figure 2:**
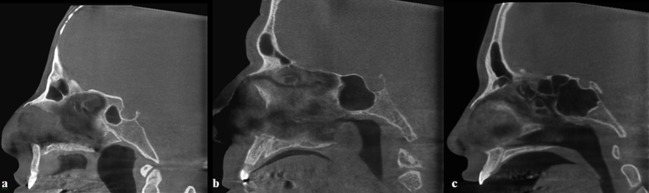
Sagittal CBCT section showing type II, III, and IV pneumatization of the sphenoid sinus  a: Type II,  b: Type III, c: Type IV

These types of sinus were best seen in the sagittal plane (Figure 2). The presence or absence of intersphenoid septa was best evaluated on both axial and coronal planes (Figure 1b). Data were evaluated by using technical properties of 90 kVp, 12.5mA, 4.55 and 3mm slice thickness in the coronal, axial, and sagittal planes. The evaluation of CBCT images was done regarding both sides of the sphenoid sinus as a separate cavity. The statistical analysis of data was done by using Chi-square test (*p*= 0.05).

## Results

A total of 103 patients were evaluated in this study. Regarding the degree of pneumatization of the sphenoid sinus, there was no case with type I (Conchal), but 2 cases (1.9%) were detected with type II (Presellar), 15 cases with type III (Sellar) (14.6%), and 86 with type IV (Postsellar) pneumatization (83.5%). Apparently, type IV was the most prevalent, affecting males more than females.

Table 1 represents the detailed data about bony anatomic variations, as well as mucosal thickening. Pneumatization of the pterygoid process and anterior clinoid process were observed in 38.9% and 33.1% of the patients, respectively. Evaluating the two sides of 103 CBCT images (206 sides); VC protrusion and FR protrusion were detected in 37.9% and 23.3% of the patients respectively. VC and FR protrusions were significantly delineated in conditions that pneumatization of PP had occurred. Carotid canal was found in 38.8% and optic canal protrusions were detected in 33% of the cases. OC protrusions was usually noticed when ACP pneumatization took place, with a statistically significant correlation between the two (*p*< 0.001). All the anatomic variations occurred more frequently in males; but it was significantly much more about pneumatization of PP, protrusion of CC, FR and VC (*p*< 0.005). Statistically significant correlations were calculated between PP pneumatization and vidian canal, foramen rotundum and carotid canal (*p*< 0.001) (Table 2). In evaluation of CBCT scans of these patients, intersphenoid septum was observed in 72 cases (69.8%).

**Table 1:** The prevalence of bony anatomic variations seen on sphenoid sinus

**Variation**	**Bilateral**	**Right side**	**Left side**	**Total**
Pneumatization				
Pterygoid Process Anterior Clinoid Process	26 (25.2%) 15 (14.6%)	6 (5.8%) 8 (7.8%)	10 (9.7%) 11 (10.7%)	42 (38.9%) 34 (33.1%)
Protrusion				
Vidian Canal Carotid Canal Optic Canal Foramen Rotundum	25 (24.3%) 26 (25.2%) 15 (4.6%) 10 (9.7%)	5 (4.9%) 4 (3.9%) 3 (2.9%) 6 (5.8%)	9 (8.7%) 10 (9.7%) 16 (15.5%) 8 (7.8%)	39 (37.9%) 40 (38.8%) 34 (33%) 24 (23.3%)
Septa	67 (65.0%)	3 (2.9%)	2 (1.9%)	72 (69.8%)

**Table 2:** The relationship between pterygoid process pneumatization and carotid canal, foramen rotundum and vidian canal protrusion

**Variation**	**Number**	**PP pneumatization**		**P value**
		**Number**	**%**	
CC Protrusion	40	26	65%	<0.001
FR Protrusion	24	42	58.8%	<0.001
VC Protrusion	39	36	85.7%	<0.001

## Discussion

The sphenoid sinus is located in the center of the cranial base. It expands in anteroposterior and lateral directions until age 10, but its full extension occurs only after adolescence.[13-14]

Various extension of the sphenoid sinus brings it in close relationship to some anatomic structures such as internal carotid artery and optic nerve.[15] Sinus and the adjacent structures in well-pneumatized cavities are separated only by a thin bony plate.[16] Prior to the development of sinus, the carotid artery, optic nerve, and vidian nerve are present; thus, concurrent with the progression of the cavity, they create irregularities in the walls of the sinus.[16] Ossama *et al.*[8] and Guldner *et al.*[17] classified the sphenoid sinus into the following four groups based on the pneumatization; Type I: conchal, Type II: presellar, Type III: sellar, and Type IV: postsellar. The least frequent type in most studies was generally conchal type sphenoid; it was found only in 2% of cases as reported in the studies performed by Levine and Clemente,[18] and Sareen *et al.*[19]

In a different study, Tan and Ong[20] investigated adult Asian cadavers and discovered the conchal type of sphenoid sinuses pneumatization to be far more frequent (28% of their specimens). In contrast, the present study found no case with this type. This type has always been regarded as a contraindication to transsphenoid approach to the sella, and consequently an unfavorable approach.[21]

A number of previous studies reported the type III (sellar type) as the most prevalent pneumatization;[8, 17, 22-23] however, in the current study, type IV was the most common of the pneumatization types.

In this study, pneumatization of the PP was 38.9% and ACP was 33.1%, which were 39.7% and 17.2%, respectively, in the study conducted by Kazkayasi *et al.*[11] Hewaidi and Omami[10] reported pneumatization of the PP in 15.3% of cases. Furthermore, Budu *et al.*[22] found this proportion to be 37.5-46.6%, and the pneumatization of ACP to be 11-29.3%.

According to the obtained results, the protrusion of optic canal was found in 33% and carotid canal in 38.8% of patients. Additionally, the protrusion of foramen rotundum and vidian canal was observed in 23.3% and 37.9% of cases, respectively. Kazkayasi *et al.*[11] reported the protrusion of OC and CC to be 4.1% and 5.2%, respectively. They also noticed the protrusion of FR in 14% of patients. The mentioned study detected statistically significant correlations between PP pneumatization and protrusion of both VC and foramen rotundum, which was in agreement with the findings of the current study (*p*< 0.001).[11] In another study, Sirikci *et al.*[12] noted 36.5% protrusion of OC, and Budu *et al.*[22] reported the protrusion of CC to be 34-93%.

Hewaidi and Omami[10] found the protrusion of OC, CC and VC to be 37.5%, 41%, and 27%, respectively. The correlations between the pneumatization of ACP and protrusion of OC was statistically significant in their study, similar to the studies of Sirikci *et al.*[12] and Kazkayasi *et al.*,[11] which conforms to the results of the present study (*p*< 0.001). Therefore, as a rule, ipsilateral anterior clinoid process pneumatization is a good indicator of optic nerve protrusion.

The internal carotid artery lies in a direct relation to the lateral wall of the sphenoid sinus and this may increase the risks during the surgery.[12] If the surgeon is unware of protrusion of the artery, even fatal hemorrhage may occur because it is almost impossible to control the bleeding of an injured internal carotid artery within the sphenoid sinus.[12] In case of protrusion of optic nerve, occurrence of injury is probable as a result of surgical trauma or as a complication of sinus disease. The risk of blindness is high if the surgeon damages the optic nerve within the sinus.[24] Sphenoid sinus infection compresses the optic canal or nerve and results in visual impairments, and also causes ischemia and venous congestion of the nerve.[25]

In the study conducted by Hewaidi and Omami,[10] the relationship between pneumatization of PP and protrusion of FR was statistically significant, which was in line with the findings of the current study (*p*< 0.001). This study also found statistically significant association between pneumatization of PP and protrusion of VC, which was in agreement with the results obtained by the above-mentioned study.

Presence of pneumatization of pterygoid process creates a great path to access the central skull base. In this condition, extended transnasal endoscopic approaches may reach the pterygoid process through the medial part of the posterior maxillary wall.[26]

According to literature, the pterygoid pneumatization provides a space containing purulent exudates which is suitable for focal infection and is associated with sinusitis. The results of the present study also support this finding. In our study, the prevalence of intersphenoid sinus septa was 4.8% unilaterally, 65% bilaterally (total 69.8%); however, Kazkayasi *et al.* reported it to be 20.6%.[11]

The present study also found the protrusion of carotid canal and pterygoid process pneumatization to be significantly correlated; whereas, it was not statistically significant in the study carried out by Sirikci *et al.*[12]

In accordance with the results, projection of vessels and nerves adjacent to the sinus wall tends to bulge in to the cavity as the whole pneumatization of the sinus increases. 

## Conclusion

A highly-pneumatized sphenoid sinus may distort the anatomic configuration. According to the present study, vital structures including the carotid, optic, and vidian canals are affected by the degree of pneumatization of the sphenoid sinus.VC and FR protrusion were seen in conditions that pneumatization of PP occurred. OC protrusion was detected when ACP pneumatization took place. In accordance with the papers, it is clear that the anatomical variations of the sphenoid sinus and its risky morphometry tend to give rise to a complexity of symptoms and potentially serious complications. In order to minimize the neural and vascular injury during the surgery, CBCT can be used in the pre-surgical evaluation of patients under consideration of endoscopic sphenoid sinus surgery. 
